# Biological Response to Meal Ingestion: Gender Differences

**DOI:** 10.3390/nu11030702

**Published:** 2019-03-26

**Authors:** Hugo Monrroy, Giulio Borghi, Teodora Pribic, Carmen Galan, Adoracion Nieto, Nuria Amigo, Anna Accarino, Xavier Correig, Fernando Azpiroz

**Affiliations:** 1 Digestive System Research Unit, University Hospital Vall d’Hebron; Centro de Investigación Biomédica en Red de Enfermedades Hepáticas y Digestivas (Ciberehd), Departament de Medicina, Universitat Autònoma de Barcelona, 08193 Bellaterra (Cerdanyola del Vallès), Spain; hmonrroy@gmail.com (H.M.); teodora.pribic@gmail.com (T.P.); cgalanhidalgo@gmail.com (C.G.); anietoruiz7@gmail.com (A.N.); aaccarino@telefonica.net (A.A.); 2 Department of Experimental Medicine (Medical Pathophysiology, Food Science and Endocrinology Section) “Sapienza” University of Rome, 00185 Rome, Italy; giulio.borghi@gmail.com; 3 Centro de Investigación Biomédica en Red de Diabetes y Enfermedades Metabólicas (Ciberdem), Metabolomics Platform, IISPV, Universitat Rovira i Virgili, 43003 Tarragona, Spain; namigo@biosferteslab.com (N.A.); xavier.correig@urv.cat (X.C.); 4 Biosfer Teslab S.L., 43201 Reus (Tarragona), Spain

**Keywords:** meal ingestion, gender differences, postprandial sensations, hedonic response, homeostatic response, metabolomic response

## Abstract

In a previous study, we demonstrated that women enjoyed and tolerated lower meal loads than men. Hence, we hypothesized that with the same meal load, their postprandial response is more pronounced than in men. We performed a randomized parallel trial in 12 women and 12 men comparing the postprandial responses to a palatable comfort meal. We measured homeostatic sensations (hunger/satiety, fullness) and hedonic sensations (digestive well-being, mood) on 10 cm scales, vagal tone by heart ratio variability and the metabolomic profile before and after meal ingestion. Gender differences were analyzed by repeated measures ANCOVA. Overall (*n* = 24), ingestion of the probe meal induced satiation, fullness, digestive well-being and improved mood (main time-effect *p* ≤ 0.005 for all). Women exhibited a more intense sensory experience, specially more postprandial fullness, than men [main gender-effect F (1, 21) = 7.14; *p* = 0.014]; hedonic responses in women also tended to be stronger than in men. Women exhibited more pronounced effects on vagal tone [main gender-effect F (1, 21) = 5.5; *p* = 0.029] and a different lipoprotein response than men. In conclusion, our data indicate that gender influences the responses to meal ingestion, and these differences may explain the predisposition and higher incidence in women of meal-related functional disorders.

## 1. Introduction

Meals induce a biological response that determines the ingestive/digestive process [1]. The response to meal ingestion depends on the characteristics of the meal and the responsiveness of the eater. The weight of gender in this context is not fully understood despite that this is key to individualized planning of diets, ranging from pure gastronomy, to health strategies and feeding of patients under various clinical conditions. 

In a previous study we evaluated the responses to a meal administered stepwise up to full satiation, and demonstrated that women enjoyed and tolerated lower meal loads than men [2]. However, the study design precluded gender comparisons of the postprandial response, due to the different meal loads administered. Furthermore, participants consumed the meal up to full satiation, whereas in normal conditions other factors determine meal consumption: people stop eating when the food is all gone (availability) or when food stops tasting good (hedonic factor) [3]. 

Since women enjoyed and tolerated lower meal loads, we hypothesized that with the same meal their postprandial response is more pronounced than in men. Hence, we compared the postprandial responses to a standard meal in women and men. Based on previous data [2], the meal load was established to induce a consistent homeostatic response with a positive hedonic dimension. To this purpose, we used an assorted, attractive meal at a load well below the level of tolerance previously established in both women and men [2]. Different parameters of the biological response to meal ingestion were measured, including sensations, physiological variables and circulating metabolites.

## 2. Material and Methods 

### 2.1. Participants

Twenty-four healthy, non-obese, non-dieting and weight-stable subjects (12 women, 12 men) without history of gastrointestinal symptoms were recruited by public advertising to participate in the study. Age was matched across gender groups; body mass index in women and men was within the respective normal range. Exclusion criteria were chronic health conditions, prior obesity, use of medications (except occasional use of NSAIDs and antihistamines), history of anosmia and ageusia, current dieting or any pattern of selective eating such as vegetarianism, alcohol abuse and use of recreational drugs. Smokers were not excluded, but participants were instructed to refrain from smoking on the study day. Absence of current digestive symptoms was verified using a standard abdominal symptom questionnaire (no symptom > 2 on a 0–10 scale). Psychological and eating disorders were excluded using the following tests: Hospital Anxiety and Depression scale (HAD), Dutch Eating Behavior Questionnaire (DEBQ - Emotional eating, External eating, Restrained eating), and Physical Anhedonia Scale (PAS). Candidates were asked whether they liked the test meals to be tested (see below) and those who did not were not included. As in previous studies, women were studied during the follicular phase of the menstrual cycle (days 5–15), in order to reduce potential variations in gut function related to the menstrual cycle [2,4].

The research was conducted according to the Declaration of Helsinki. The protocol for the study had been previously approved by the Institutional Review Board of the University Hospital Vall d’Hebron, and all participants gave written informed consent.

### 2.2. Experimental Design

This study was a single-center, parallel randomized study performed between June and September 2018. The study investigated the effect of gender on the responses to meal ingestion. The primary outcome was fullness sensation in response to the test meal. Men and women were studied in random order determined by a computer-generated randomization list. The study protocol was registered with the ClinicalTrials.gov NCT03758482. All co-authors had access to the study data and reviewed and approved the final manuscript.

### 2.3. General Procedure

Each participant was studied once. Participants were instructed to refrain from strenuous physical activity the day prior to the study, to consume only a standard breakfast (coffee with semi-skimmed milk and biscuits; 181 Kcal, 5 g lipids, 27 g carbohydrate, 7 g protein) at home after overnight fast and to report to the laboratory, where the probe meal was administered 4 h after breakfast. Studies were conducted with participants sitting alone with one investigator in a quiet, isolated room. Perception was measured before, during and after meal ingestion. Heart rate variability (see below) and blood pressure (M6AC, Omron, Kyoto, Japan) were measured during baseline, early postprandial period and 30 min and 60 min after ingestion. Body temperature was measured using a digital thermometer (DT-502EC, A&D Instruments, Oxford, UK) before and 60 min after ingestion. Venous blood samples were taken immediately before and 30 min after the probe meal.

### 2.4. Probe Meal and Procedure

The probe meal consisted of: 50 g duck fatty liver (Foie Gras Entero de Pato «Mi-cuit», El Corte Inglés, Larrabezua, Spain), 15 g toasts (Mini Tostas, Bimbo, Barcelona, Spain), 50 g cheese (Queso Emmental, El Corte Inglés, Madrid, Spain), 25 g potato chips (Patatas Fritas Clásicas, LAY’S, Vitoria, Spain), 10 g salted peanuts (Cacahuetes Fritos, Borges, Tarragona, Spain) and 140 mL soft drink (Coca-Cola Clásica, Coca-Cola, Madrid, Spain) (Table 1). The ingestion rate was standardized in three steps: (1) 10 g duck fatty liver, 3 g toast and 10 g peanut in 2 min; (2) 40 g duck fatty liver, 12 g toast and 25 g potato chips in 6 min; (3) 50 g cheese in 2 min; soft drink (140 mL) was drunk at demand during the ingestion period (total 10 min). The eating rate was decided based on preliminary studies searching for a pleasant rate.

### 2.5. Perception Measurements 

Five 10 cm scales graded from −5 to +5 were used to measure: (a) meal wanting (negative/eager), (b) meal liking (very disagreeable/very agreeable, (c) hunger/satiety (extremely hungry/completely satiated), (d) digestive well-being (extremely unpleasant sensation/extremely pleasant sensation) and (e) mood (negative/positive); two additional 10 cm scales graded from 0 (not at all) to 10 (very much) were used to measure: (f) abdominal bloating-fullness, and (g) discomfort-pain. Subjects received standard instructions on how to fill-out scales [5]. The wanting scale was only scored at presentation of meal. The liking scale was only scored at the end of ingestion. The rest of the scales, were scored at 5 min intervals 10 min before and 20 min after ingestion and at 10 min intervals up to 60 min after the probe meal. 

### 2.6. Assessment of Heart Rate Variability

Continuous cardiac interbeat intervals (IBI) for each subject were recorded using a lightweight device (Bittium Faros 360°, Mega Electronics, Kuopio, Finland) from 5 surface electrodes at 500 Hz sampling rate. Heart rate variability (HRV) was assessed over 5 min recording periods during baseline, early postprandial period and 30 min and 60 min after ingestion.

HRV analysis of the exported data was performed on computer using dedicated HRV software (Kubios Premium ver. 3.1.0) as previously described [6]. Prior to HRV computation all IBI data were visually inspected for correctness and then underwent automatic artifact correction. HRV spectra were calculated by autoregressive transformation. High-frequency power data (0.15–0.40 Hz) are reported as normalized units [7,8]. Respiratory rate was calculated using an ECG-derived respiration software within the HRV analysis package [9].

### 2.7. Analytical Procedures

Once extracted, venous blood samples were immediately placed in ice. After completing the study, the samples were centrifuged for 15 min at 4 °C at 1500× *g* to separate blood components. Using 1 mL pipettes, samples of plasma and serum were placed in Eppendorf tubes and stored at −40 °C.

Routine laboratory techniques were used to measure insulin (AU5800 Spectrophotometry, Beckman Coulter, Brea, CA, USA), cortisol (Advia Centaur XP Immunoassay, Siemens, Tarrytown, NY, USA) and ACTH levels (Liaison CLIA, Diasorin, Saluggia, Italy).

The metabolomic analysis was performed using nuclear magnetic resonance (NMR), as previously described [10,11]. In brief, the concentration of the three different classes of lipoproteins (VLDL, LDL, and HDL), and their composition (content of cholesterol, triglycerides and large, medium and small particles) were determined [12]. A target set of 14 low molecular weight metabolites (LMWMs) was identified and quantified in the 1D Carr-Purcell-Meiboom-Gill spectra using Dolphin [13]. Each metabolite was identified by checking for all its resonances along the spectra and then quantified using line-shape fitting methods on one of its signals [14]. Validation of metabolite identification was assisted by STOCSY [15].

### 2.8. Statistical Analysis 

Statistical analysis was performed using the Stata Software for Windows, (StataCorp. 2017. Stata Statistical Software: Release 15. College Station, TX: StataCorp LLC) and MetaboAnalyst 4.0 [16].

Based on previous data [2], it was estimated that a sample size of 10 subjects per group would allow to detect gender differences in postprandial fullness with 80% power and 5% significant threshold.

In each group, means and SE of the measured variables were calculated. The Kolmogorov-Smirnov test was used to check the normality of data distribution. Parametric normally distributed data were compared by Student’s *t*-test for paired or unpaired data; otherwise, the Wilcoxon signed rank test was used for paired data, and the Mann-Whitney *U* test was used for unpaired data. The association of parameters was evaluated using the linear regression analysis.

Temporal responses to meal ingestion were analyzed using one-way ANOVA for repeated measurements (10 min pre and 60 min postprandial period); when the ANOVA was significant, post hoc comparisons between time-points were performed applying the Sidak multiple comparison correction procedure. Comparison between groups (men vs. women) were performed with a repeated measures ANCOVA (dependent variable: postprandial sensations scores; between and within subject’s factors: gender and time, respectively; covariate: premeal scores) [17].

Multivariate discriminant analysis of metabolomic data (standardized concentrations) was performed using an unsupervised classification by Principal Component Analysis (PCA) and supervised orthogonal partial least squares discriminant analysis (OPLS-DA). 

Differences were considered significant at a *p*-value < 0.05.

## 3. Results

### 3.1. Demographics

Participants were 21–36 years age range without differences between women and men. Body weight and height were 57 ± 2 kg and 165 ± 1 cm in women, and 77 ± 2 kg and 180 ± 2 cm, respectively, in men (*p* < 0.001 for both). Body mass index range was 18.5–23.9 kg/m^2^ in women and 21.2–24.9 kg/m^2^ in men. All participants had normal bowel habit and scored HAD, PAS and DEBQ within the normal range. No significant difference in the proportion of smokers was detected between groups (3/12 in women and 5/12 in men; *p* = 0.667). All participants completed the studies and were included for analysis.

### 3.2. Baseline Conditions 

**Figure 1:**
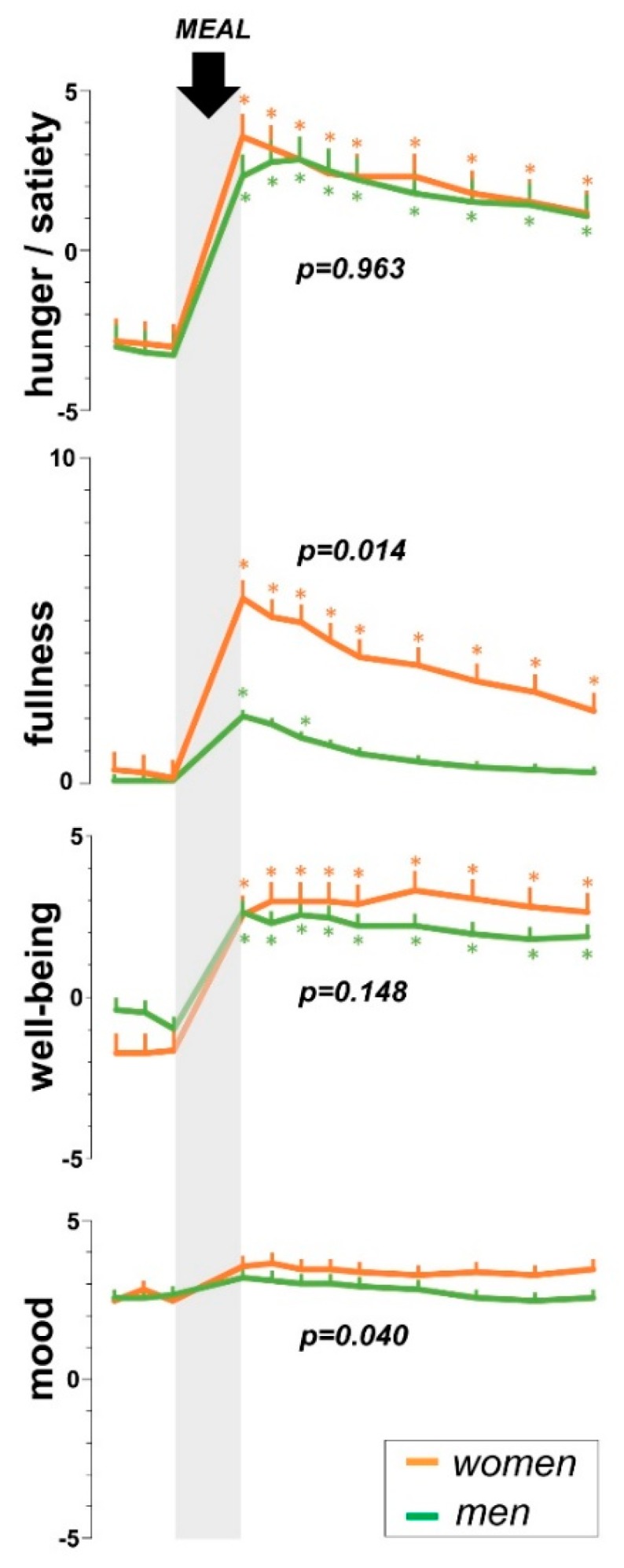
Postprandial experience in women and men. Main gender-effect by repeated measures ANCOVA shown; dependent variable: postprandial scores; covariate: pre-meal scores. Temporal responses to meal ingestion analyzed by one-way ANOVA for repeated measures; asterisks indicate significant differences from premeal values by post-hoc comparisons (*p* < 0.05 applying the Sidak correction procedure for multiple comparisons). Postprandial sensations tended to be higher in women than in men, but the differences in satiety and digestive well-being were blurred because the scores were near the top of the scales in both groups.

Before the probe meal (baseline fasting period), participants reported hunger with no symptoms (fullness/bloating, discomfort/pain) and positive mood (Figure 1). 

### 3.3. Sensory Responses to Meal Ingestion

The probe meal resulted attractive and before ingestion participants reported positive wanting score (2.7 ± 0.3 score in women and 3.7 ± 0.2 score in men; *p* = 0.008). All participants finished the meal at the same ingestion rate. Participants liked the probe meal and at the end of ingestion reported positive liking scores (2.3 ± 0.4 score in women and 2.9 ± 0.2 score in men; *p* = 0.126). 

Overall (*n* = 24), ingestion of the probe meal induced satiation, fullness, digestive well-being and improved mood (main time-effect *p* ≤ 0.005 for all); these sensations gradually decayed during the postprandial period. Postprandial satiety scores were high in both groups, and hence, no gender differences were detectable (main gender-effect F (1, 21) = 0.0; *p* = 0.963) (Figure 1). However, a clear difference in fullness sensations was observed: fullness sensation was mild and transient in men, but significantly higher and more persistent in women (main gender-effect F (1, 21) = 7.14; *p* = 0.014). By multiple linear regression analysis, fullness sensation correlated with gender (β = −0.83; *p* = 0.006) but not with body weight (β = 0.17; *p* = 0.539).

Well-being scores were somewhat higher in women than in men (Figure 1); similarly to satiety, meal ingestion induced strong and persistent effects on digestive well-being that blurred gender differences: the difference was detected comparing the area under the curve (*p* = 0.020) but not by ANCOVA (main gender-effect F (1, 21) = 2.25; *p* = 0.148). In both groups the effect of meal ingestion on mood was mild, but significantly higher in women than in men (main gender-effect F (1, 21) = 4.8; *p* = 0.040).

### 3.4. Physiological Responses

**Figure 2:**
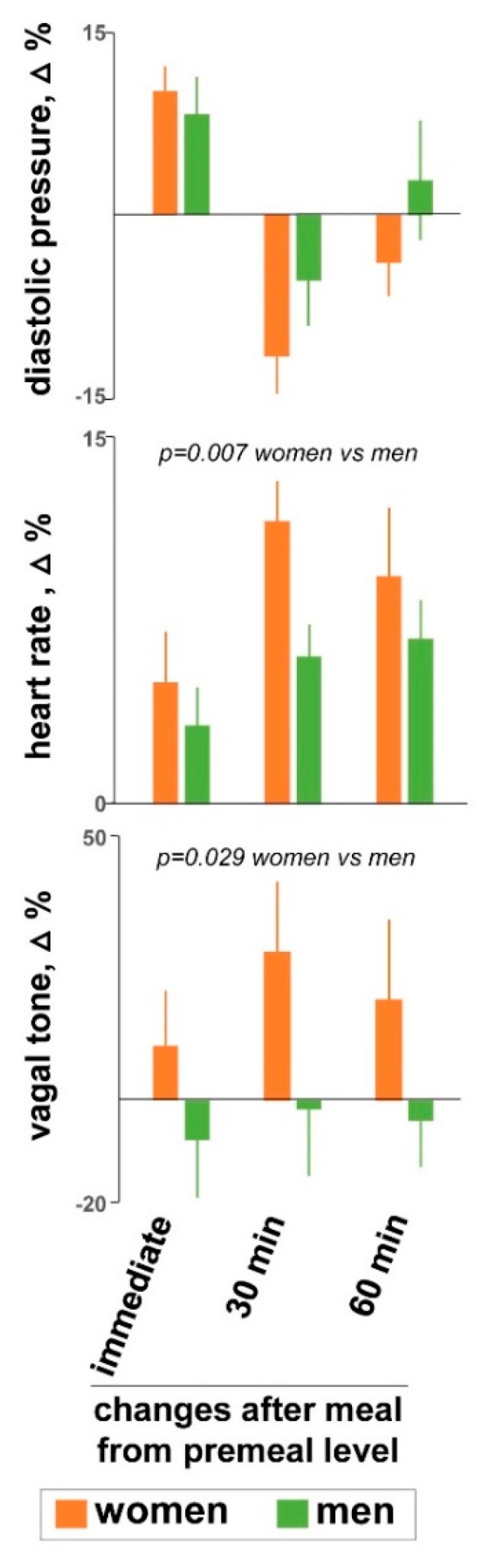
Physiological responses to probe meal. Data are postprandial changes from premeal baseline (mean ± SE). Overall, meal ingestion was associated with an increase in diastolic blood pressure, heart rate and vagal tone (main time-effect *p* ≤ 0.003 for all; *n* = 24). As compared to men, women exhibited more pronounced effects on heart rate and vagal tone (by ANCOVA).

Overall (*n* = 24), meal ingestion was associated with changes in diastolic blood pressure (main time-effect *p* = 0.004), heart rate (main time-effect *p* = 0.003), and vagal tone (main time-effect *p* = 0.004), without changes in systolic blood pressure (main time-effect *p* = 0.697) and respiratory frequency (main time-effect *p* = 0.253) (Figure 2). Body temperature increased by 0.21 ± 0.09 °C (*p* = 0.044). As compared to men, women exhibited more pronounced effects on heart rate (main gender-effect F (1, 21) = 13.4; *p* = 0.007) and vagal tone (main gender-effect F (1, 21) = 5.5; *p* = 0.029), but no other gender differences were detected.

### 3.5. Hormonal Response

Meal ingestion increased circulating levels of insulin (*p* < 0.001) and decreased ACTH (*p* = 0.035) without changes in cortisol (0.362); no gender differences in the hormonal responses to the probe meal were detected.

### 3.6. Metabolomic Response

#### 3.6.1. Low-Molecular Weight Metabolites

Overall (*n* = 24) meal ingestion induced changes in the profile of circulating metabolites. Specifically, alanine (*p* = 0.009), glutamine (*p* = 0.009), isoleucine (*p* = 0.007), histidine (*p* = 0.040) and valine (*p* = 0.024) increased and 3-hydroxybutirate decreased (*p* = 0.029). No gender differences were observed.

#### 3.6.2. Lipoprotein Profile

**Figure 3:**
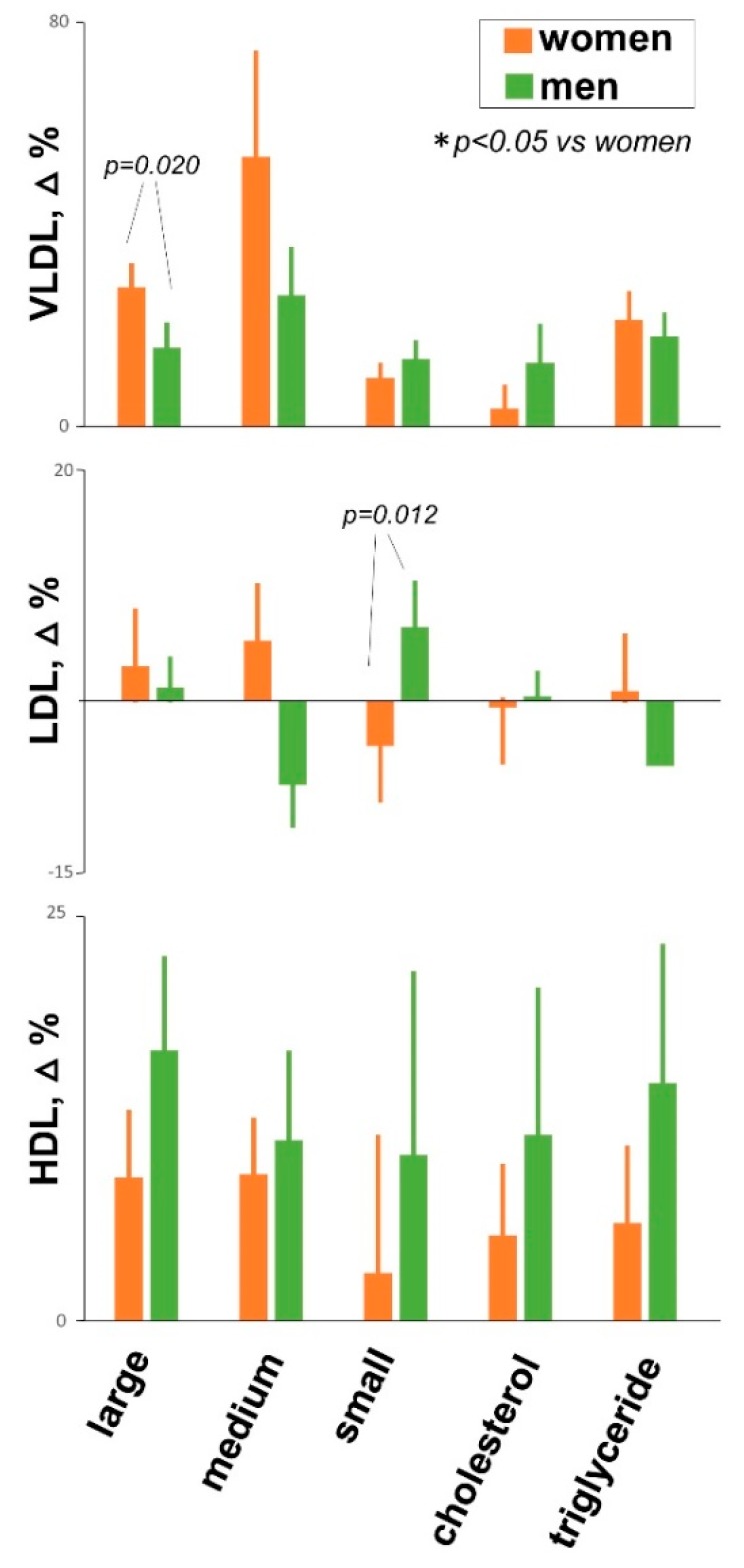
Lipidic response to meal ingestion. Meal ingestion induced changes in the profile of plasma lipoproteins with differences between women and men. Dara are means ± SE.

Meal ingestion induced a change in the profile of plasma lipoproteins with some differences between women and men (Figure 3). Using an unsupervised PCA and OPLS-DA model women and men exhibited different distribution (data not shown).

## 4. Discussion

Our study shows gender differences in the biological responses to meal ingestion. Women exhibited a more profound sensory experience than men, involving both homeostatic and hedonic sensations, associated to differences in vagal tone and lipid metabolism.

With the same meal, women experienced stronger homeostatic sensations, in particular more postprandial fullness, than men. No differences in satiety were detected, and this was conceivably related to a saturation effect, because satiety scores were close to the top of the scales in both groups. In general, satiation precedes fullness in response to meal ingestion and satiety increases more steeply as a function of the meal load than fullness [1]. Hence, detection of gender differences depends on the meal load: a smaller meal would induce less satiety in men uncovering gender differences, whereas a larger meal would also drive fullness scores to the top of the scales both in women and men blurring the difference.

Hedonic responses in women also tended to be more pronounced than in men. Previous studies showed that the relation of homeostatic to hedonic sensations is bimodal: with meal loads in the lower range homeostatic and hedonic sensations are directly related, and both increase as a function of the meal load; with larger meal loads the relation becomes inverse, and while homeostatic sensations steadily increase, hedonic sensations decrease down to a minimum at the level of full satiation [2]. This phenomenon is similar in women and men, but in women the curve is shifted to the left, so that they experience both the well-being peak (maximal satisfaction), as well as full satiation with lower meal loads than men [2]. Conceivably, the meal load in the present study was close to the yield point in women, who experienced strong homeostatic sensations with a potent hedonic dimension, whereas men would have enjoyed a larger meal load.

Several factors may play a role in these gender differences. In the first place, body weight in women was smaller than in men. Furthermore, women have lower metabolic rates and energy requirements than men [18,19,20] and these differences persist after controlling for body mass [18]. Theoretically, more fullness with the same meal load in women could be related to smaller gastric capacity, which would seem plausible considering their smaller body size; however, the size of the stomach is similar in women and men. Indeed, measuring gastric compliance by means of an air-filled flaccid bag connected to a barostat, it was shown that during basal conditions (empty stomach in the fasting state) at fixed (standardized) intragastric pressure levels, intraluminal volume (gastric capacity) was similar in women and men [21]. These studies also measured the sensations induced by gastric distension, and interestingly, the same pressure (and volume) levels induced more intense perception in women. These data indicate that despite the similar size, the stomach in women is more sensitive and they perceive and tolerate smaller intraluminal loads than men. Lower tolerance in women has been also reported using drink tests that administer water or a nutrient drink at a fixed ingestion rate up to the level of discomfort [22,23,24]. 

The gastric response to a meal is under vagal control [25,26]. During fasting the stomach is contracted and meal ingestion triggers a vagal reflex that produces a gastric relaxation to accommodate the meal [27]. Studies using the gastric barostat have shown that despite the lack of differences in gastric compliance during fasting, the gastric accommodation reflex is more prolonged in women than in men [21], and this may be related to the more pronounced vagal tone response to the meal in women observed in our study, although admittedly, measurements of vagal tone based on heart rate variability are rather unspecific.

In a previous study we normalized the meal load to satiation levels; and in accordance to the present data, men ate more [2]. Under these conditions they exhibited a more pronounced response in the low-molecular weight metabolites profile, but it was not clear whether and to what extent this difference was related to gender or to the meal load [2]. The current study helps to interpret these data by showing that at the same meal load, low-molecular weight metabolites were similar in both groups, indicating that the meal load rather than gender determines the differences. Interestingly, the situation is different in relation to lipid metabolism, where gender differences were apparent independently of the meal load. These differences in lipoproteins metabolism are in line with previous observations [28,29] and may be related to gender-specific metabolic activity patterns [19,20,30]. 

## 5. Conclusions

Gender differences in the biological responses to meal ingestion may help to explain the specific eating behaviour in women and men; indeed, in accordance with our data, it has been previously shown that women consume less energy per kg lean mass [31,32], and on standard diet adjusted to meet individual energy requirements, women exhibit less appetite than men [19,20]. From a practical point of view, gender differences have to be considered when testing the responses to meal ingestion, and this is particularly important in patients with meal-related symptoms. Furthermore, gender differences may be involved in the higher predisposition and incidence in women of functional disorders triggered by meal ingestion [33,34].

## Figures and Tables

**Table 1:** Probe meal.

	Total (g)	Total (kcal)	FAT (g)	PROT (g)	CHO (g)
Fatty liver duck *	50	265.5	27.5	4.0	0.5
Toast	15	60.7	0.7	1.8	11.4
Cheese	50	173.5	13.5	13.0	0.0
Potato chips	25	127.7	7.8	1.5	12.3
Peanuts	10	63.8	5.3	2.8	1.0
Drink	140	58.8	0.0	0.0	14.8
Probe meal	290	750.0	54.8	23.1	40.0

PROT: proteins, CHO: carbohydrates. * Foie gras mi-cuit.
